# Improving *Clostridioides difficile* Infectious Disease Treatment Response via Adherence to Clinical Practice Guidelines

**DOI:** 10.3390/antibiotics13010051

**Published:** 2024-01-04

**Authors:** Dalia Adukauskienė, Rytis Mickus, Asta Dambrauskienė, Tomas Vanagas, Agnė Adukauskaitė

**Affiliations:** 1 Medical Academy, Lithuanian University of Health Sciences, 44307 Kaunas, Lithuania; dambrauskienea@gmail.com (A.D.); tomas.vanagas@kaunoklinikos.lt (T.V.); 2 Department of Cardiology and Angiology, University Hospital of Innsbruck, 6020 Innsbruck, Austria; agne.adu@gmail.com

**Keywords:** antibiotic optimization, Charlson Comorbidity Index (CCI), *Clostridioides difficile* infectious disease (CDID), precipitating antibiotics, concomitant antibiotics, Intensive Care Unit, criteria for severe *C. difficile* infection, metronidazole, vancomycin

## Abstract

*Clostridioides difficile* (*C. difficile*) is a predominant nosocomial infection, and guidelines for improving diagnosis and treatment were published in 2017. We conducted a single-center, retrospective 10-year cohort study of patients with primary *C. difficile* infectious disease (CDID) at the largest referral Lithuanian university hospital, aiming to evaluate the clinical and laboratory characteristics of CDID and their association with the outcomes, as well as implication of concordance with current Clinical Practice Guidelines. The study enrolled a total of 370 patients. Cases with non-concordant CDID treatment resulted in more CDID-related Intensive Care Unit (ICU) admissions (7.5 vs. 1.8%) and higher CDID-related mortality (13.0 vs. 1.8%) as well as 30-day all-cause mortality (61.0 vs. 36.1%) and a lower 30-day survival compared with CDID cases with concordant treatment (*p* < 0.05). Among cases defined by two criteria for severe CDID, only patients with non-concordant metronidazole treatment had refractory CDID (68.8 vs. 0.0%) compared with concordant vancomycin treatment. In the presence of non-concordant metronidazole treatment for severe CDID, only cases defined by two severity criteria had more CDID-related ICU admissions (18.8 vs. 0.0%) and higher CDID-related mortality (25.0 vs. 2.0%, *p* < 0.05) compared with cases defined by one criterion. Severe comorbidities and the continuation of concomitant antibiotics administered at CDID onset reduced (*p* < 0.05) the 30-day survival and increased (*p* = 0.053) 30-day all-cause mortality, with 57.6 vs. 10.7% and 52.0 vs. 25.0%, respectively. Conclusions: CDID treatment non-concordant with the guidelines was associated with various adverse outcomes. In CDID with leukocytes ≥ 15 × 10^9^/L and serum creatinine level > 133 µmol/L (>1.5 mg/dL), enteral vancomycin should be used to avoid refractory response, as metronidazole use was associated with CDID-related ICU admission and CDID-related mortality. Severe comorbidities worsened the outcomes as they were associated with reduced 30-day survival. The continuation of concomitant antibiotic therapy increased 30-day all-cause mortality; thus, it needs to be reasonably justified, deescalated or stopped.

## 1. Introduction

*Clostridioides difficile* (*C. difficile*) is an anaerobic Gram-positive, spore-forming and toxin-producing microorganism [1]. *C. difficile* remains one of the most common opportunistic pathogens to cause nosocomial diarrhea associated with the use of antibiotics [1]. Despite the use of antibiotics upon infection being lifesaving, if administered inappropriately, it is associated with unfavorable harm [2]. Previous antibiotic therapy is the main risk factor for *C. difficile* infectious disease (CDID), usually manifesting as colitis and sometimes as enteritis; it may even present with a septic condition [3,4,5,6,7,8]. Significant comorbidities are associated with more frequent hospital admissions, and these patients are at risk of CDID, as it tends to develop in hospitalized cases [4,9]. The development of CDID is associated with mortality, especially in Intensive Care Unit settings [10,11]. The use of an elevated leucocyte count (≥15 × 10^9^/L) or/and high creatinine serum level (>133 µmol/L) as criteria, as recommended by Clinical Practice Guidelines, distinguishes severe from non-severe infection, which may be related to significantly different outcomes, and this may help the immediate optimization of the treatment for the purpose of better outcome control [12,13]. A severe form of CDID is diagnosed if either one or both of the previously mentioned criteria are detected [12]. However, to our knowledge, no emphasis has been put on the differences between patients meeting two criteria (“very severe” form) and just one criterion for CDID severity. The prompt discontinuation of the causative antibiotics is important because studies have shown that continuation (established as concomitant antibiotics for the treatment of concurrent infection) can be a significant risk factor for poorer outcomes [14,15,16].

Though CDID is being triggered by antibiotics, particular antibiotic therapy is a standard to control this disease [12]. Enteral vancomycin has been reported to have a more clinically favorable effect in reducing the recurrence and mortality of CDID when used for the treatment of the primary episode, especially in severe form, compared to metronidazole [17,18,19,20]. The 2017 Clinical Practice Guidelines developed for the improvement of CDID diagnosis and treatment recommend the use of vancomycin as an optimal choice over the previously used metronidazole, with the intent to improve control of this infection [12]. Metronidazole could still be used in non-severe cases or in cases of vancomycin unavailability [12,18].

Aiming to evaluate the significance of concordance with Clinical Practice Guideline recommendations in relation to the endpoints, we formulated the following hypothesis: the response to treatment and certain outcomes of primary CDID depend on concordance with the treatment recommendations of the guidelines, which must remain the focus of clinicians. Thus, the primary predictions of this study were as follows: CDID treatment non-concordant with the guidelines results in worse outcomes; the response to CDID treatment also produces worse outcomes in cases of severe CDID defined by two (both) diagnostic criteria and treated using non-concordant treatment. The secondary objectives were to associate the comorbidities as well as continuity of concomitant antibiotics with the 30-day survival and 30-day all-cause mortality when treatment was concordant with the guidelines. With the purpose of estimating whether antibiotic optimization has an impact on the rate of CDID, we investigated the dynamics of the use of CDID precipitating antibiotics also antibiotics used for primary CDID treatment, before and after following the guidelines.

## 2. Materials and Methods

We conducted a single-center, retrospective cohort study at the largest university-affiliated hospital in Lithuania (approvals no. BEC-LSMU(R)-14 and no. BEC-MF-869 for the collection and analysis of materials used for the study were obtained from the Kaunas Regional Biomedical Research Ethics Committee). Due to the observational nature of the study, the need for written consent was waived. 

Inclusion criteria: 1. patients aged ≥ 18 years; 2. diarrhea ≥ 24 h (≥3 defecations per day and/or increased stool volume of >200 g per day); 3. *C. difficile* A and/or B toxins in stool detected via enzyme immunoassay analysis; 4. primary episode of CDID [12,21].

Exclusion criteria: 1. patients with case histories unavailable for retrieval; 2. patients with the absence of documented clinical CDID characteristics; 3. patients with a confirmed episode of recurrent CDID only; 4. patients with a lethal outcome on the first day after CDID diagnosis.

Recurrent CDID was defined as recurring diarrhea with the detection of *C. difficile* A and/or B toxins in stool 2–8 weeks following the initial CDID episode [12].

**Figure 1:**
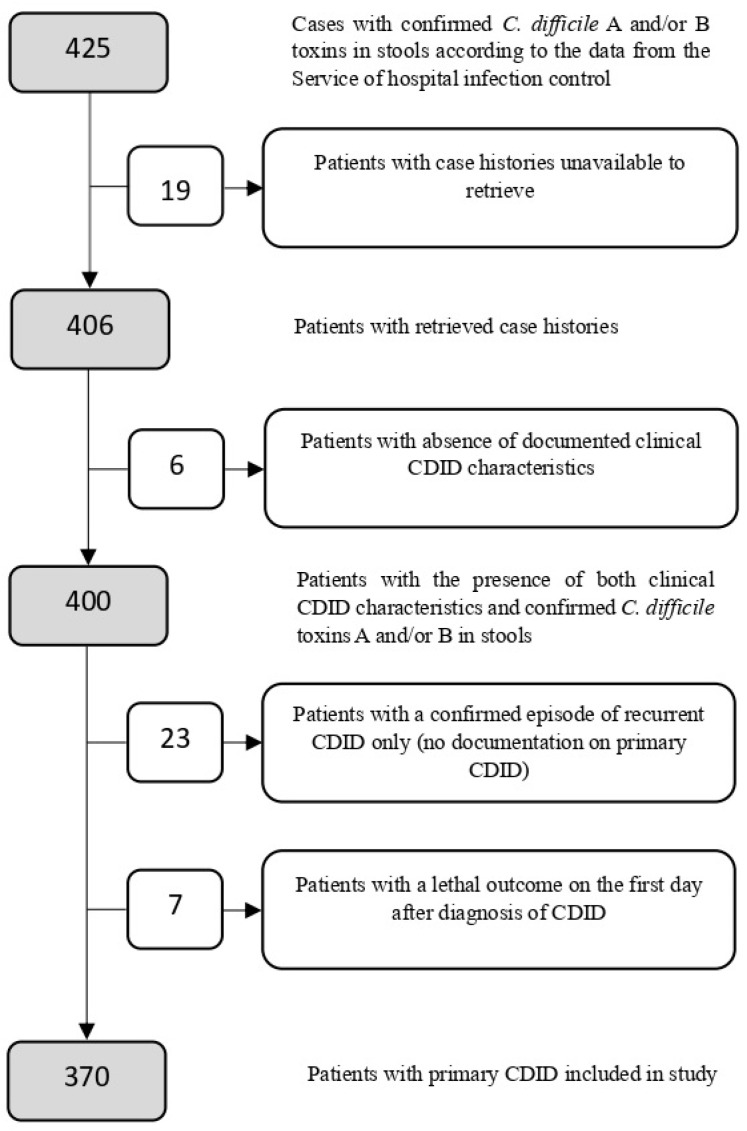
Detailed flowchart of patients enrolled in the study.

The inclusion and exclusion criteria for the total number of patients are presented in Figure 1.

Thus, patients with just clinical diagnosis and unconfirmed A and/or B toxins in stool at diagnosis of CDID were not included in the study [12].

The entire sample, covering the 10-year period from 1 January 2011 to 31 December 2020, was analyzed. The period studied was divided into two intervals: before (Group 1, from 2011 to 2016) and after (Group 2, from 2017 to 2020) integrating the 2017 Clinical Practice Guidelines for CDID [12]. The data collection period extended from 17 February 2017 to 15 July 2022. 

The hospital departments (medical, surgical, Intensive Care Unit) where CDID was diagnosed were identified.

Patients were considered immunocompromised in the case of the following: hereditary or primary immunodeficiency, malignancy, diabetes mellitus, detection of human immunodeficiency virus infection, harmful substance abuse, nutritional deficiency, hepatic insufficiency and use of immunosuppressants or antineoplastic agents.

Leukocytes ≥ 15 × 10^9^/L and/or serum creatinine level > 133 µmol/L (equivalent to >1.5 mg/dL) were used as criteria to diagnose clinically severe CDID as recommended by the 2017 Clinical Practice Guidelines [12]. Severe comorbidities were regarded as the Charlson Comorbidity Index score of ≥5 [22,23,24,25]. A precipitating antibiotic was defined as one the patient was exposed to during a period of 12 weeks prior to CDID (antibiotic therapy as a risk factor for CDID) [26]. A concomitant antibiotic was regarded as one of which the use was continued for ≥3 days after the diagnosis of CDID due to the need to treat other (non-*C. difficile*) infections [27].

The recommended treatment was considered the selection and administration of either vancomycin or metronidazole for non-severe CDID, and the administration of only vancomycin for severe CDID [16]. Non-recommended treatment was defined as only metronidazole given for the treatment of severe CDID or no antibiotics used for the treatment of CDID [12]. An appropriate initial treatment was considered administration via the enteral route, consisting of a metronidazole dose of 500 milligrams three times a day and a vancomycin dose of 125–500 milligrams four times a day continued for ≥3 days [12,28]. CDID treatment was considered concordant with the guidelines when aligning with the recommendations (selection of antibiotic compatible with the guidelines) appropriately (route, dose and duration).

Group M was defined as cases with severe CDID and (non-concordant) enteral metronidazole treatment, and Group V as cases with severe CDID and (concordant) enteral vancomycin treatment.

A patient was censored when the outcome within 30 days of CDID diagnosis was inestimable due to discharging from the hospital. A 30-day follow-up was used to assess the 30-day all-cause mortality.

Refractory CDID (treatment failure) was determined as persisting clinical symptoms (diarrhea) for ≥3 days after the onset of treatment [28].

We defined the outcomes of CDID as CDID-related Intensive Care Unit admission, CDID-related mortality, 30-day survival and 30-day all-cause mortality. CDID-related Intensive Care Unit admission was regarded as one occurring with an active clinical manifestation of CDID (diarrhea) on the day or following ones after the diagnosis of CDID [29,30]. CDID-related fatal outcome was determined by medical death certificates when clinical symptoms of CDID remained persistent [30].

Antibiotic optimization was defined as (1) a reduction in the use of CDID precipitating broad-spectrum antibiotics and (2) an increase in concordant vancomycin use for the treatment of primary CDID in dynamics.

To accomplish our objectives, we designed a query according to the Population, Intervention, Control, Outcome (PICO) framework (Supplementary Material S1. PICO chart for the study objectives): 1. Is concordant treatment (in comparison with non-concordant) associated with the outcomes for all CDID cases? 2. In cases with non-severe CDID, is concordant treatment (as compared with non-concordant treatment) associated with CDID-related adverse outcomes? 3. In cases with severe CDID meeting two (both) severity criteria, does vancomycin (as compared with metronidazole) improve the response to initial treatment? 4. In cases with severe CDID approached with non-concordant metronidazole treatment, are more severe cases defined by two severity criteria (as compared with by one criterion) associated with CDID-related adverse outcomes? 5. In cases of severe and non-severe CDID and concordant treatment, are severe comorbidities (in comparison with non-severe comorbidities) associated with the outcomes? 6. In cases of severe and non-severe CDID and concordant treatment, is the continuation of concomitant antibiotics (as compared with discontinuation) associated with adverse outcomes? 7. In all cases of CDID, is antibiotic optimization associated with the CDID rate?

In our study, we followed the STROBE checklist guidelines (Supplementary Material S2. STROBE checklist) to optimize the structure of the gathered data for a comprehensible presentation of the results [31].

### Statistical Analysis

The SPSS 23.0 software package was used for data analysis. In the presence of non-normal distribution, quantitative variables were expressed as a median (Q1; Q3). The Shapiro–Wilk test was applied to test the distribution of quantitative variables. Relative frequencies of qualitative variables were expressed as proportions (percentages), and Pearson’s chi square as well as Fisher’s exact tests were used to compare their differences between the groups. The Mann–Whitney U test was applied to compare the means between the groups. Binary logistic regression analysis was used to predict the relationship between independent variables and dependent dichotomous variables, and the results were displayed as odds ratios with 95% confidence intervals. The Kaplan–Meier model was employed to estimate the effect of qualitative variables on survival over time, and the log-rank, Breslow and Tarone–Ware tests were used to compare the groups. All statistical tests were two-sided, statistical significance was set at *p* < 0.05 and borderline (clinical) significance was considered *p* = 0.05–0.1.

The G*Power 3.1.9.4. software was used to estimate the power of the study.

## 3. Results

### 3.1. General Characteristics

The study enrolled a total of 370 patients. The minimal sample size of the study was estimated to be n = 197 patients to achieve a study power of 0.8 and effect size of 0.2. The characteristics of the study sample are presented in Table 1.

**Figure 2:**
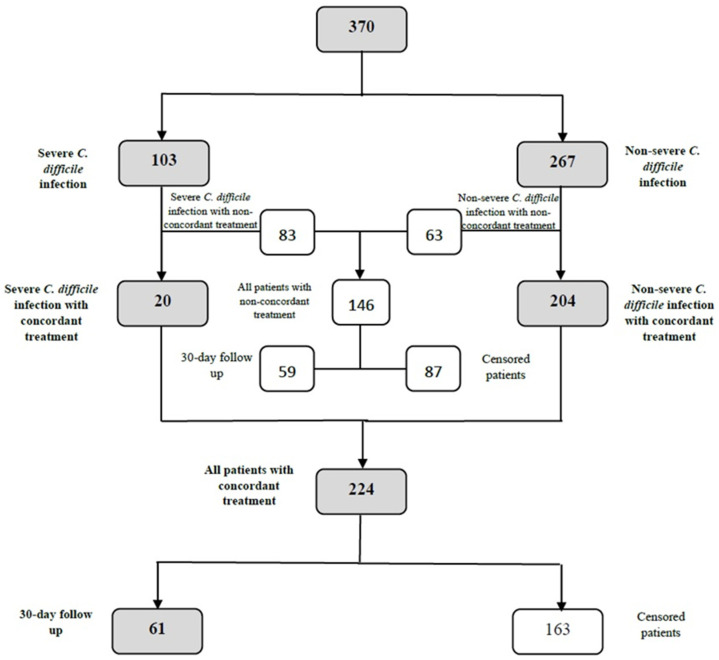
Detailed flowchart of patient differentiation in accordance with the provided treatment and eligibility for the 30-day follow-up.

The patient selection according to the treatment is presented in Figure 2.

### 3.2. All Cases of CDID

Among the 370 study patients, CDID-related ICU admission in the case of treatment in concordance with the guidelines was n = 4/224 (1.8%) vs. n = 11/146 (7.5%) for the non-concordant cases, and the incidence of CDID-related mortality was n = 4/224 (1.8%) vs. n = 19/146 (13.0%), respectively, *p* = 0.008, *p* = 0.000016.

**Figure 3:**
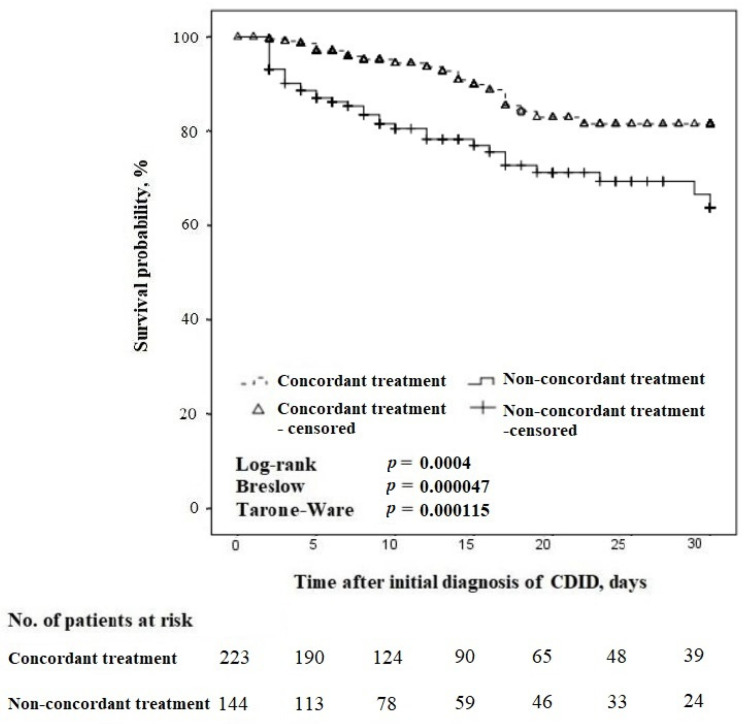
Kaplan–Meier survival curves for time to death in concordant vs. non-concordant treatment in patients with CDID (censored at 30 days).

The 30-day survival was associated with concordance with the guidelines, regardless of the difference in CDID severity (Figure 3).

In the 30-day follow-up, 120 patients were included (Figure 2). The 30-day all-cause mortality was n = 22/61 (36.1%) in cases of concordant treatment, compared with n = 36/59 (61.0%) for non-concordant treatment, *p* = 0.01.

### 3.3. Cases of Non-Severe CDID

In the 267 non-severe cases, CDID-related ICU admission when treatment was in concordance with the guidelines compared to non-concordant cases was n = 3/204 (1.5%) vs. n = 5/63 (7.9%), and CDID-related mortality was n = 3/204 (1.5%) vs. n = 7/63 (11.1%), respectively, *p* = 0.02, *p* = 0.002.

### 3.4. Cases of Severe CDID

We estimated a total of n = 103/370 (27.8%) patients to have severe CDID; among them, n = 83/103 (80.6%) received treatment non-concordant with the guidelines.

Upon comparing concordant vs. non-concordant treatment in severe CDID cases, no statistically significant difference in outcomes was estimated.

In all cases with severe CDID with treatment non-concordant with the guidelines, n = 65/83 (78.3%) patients were administered enteral metronidazole (Group M). The remainder (n = 18) received the following: n = 5/83 (6.0%) intravenous vancomycin or metronidazole, n = 3/83 (3.6%) improper dose of enteral vancomycin and 3/83 (3.6%) inadequate initial duration of treatment. Finally, n = 7/83 (8.4%) had no documented treatment for CDID.

A total of n = 3/20 (15.0%) patients from Group V had two severity criteria for CDID, and among them, none had refractory CDID compared with n = 11/16 (68.8%) of Group M patients with two severity criteria and refractory CDID.

A total of n = 49/65 (75.4%) patients from Group M met one severity criterion (leukocytes ≥ 15 × 10^9^/L or creatinine > 133 µmol/L) and n = 16/65 (24.6%) two severity criteria. Among the patients from Group M meeting one severity criterion, none required CDID-related Intensive Care Unit admission, as compared with n = 3/16 (18.8%) Group M patients meeting two severity criteria. In Group M, the CDID-related mortality rate was n = 1/49 (2.0%) among patients defined by one severity criterion, compared with n = 4/16 (25.0%) Group M patients defined by two criteria, *p* = 0.01.

### 3.5. All CDID Cases with Concordant Treatment

The relations of multiple covariates to 30-day all-cause mortality in patients administered treatment concordant with the guidelines are presented in Table 2.

**Figure 4:**
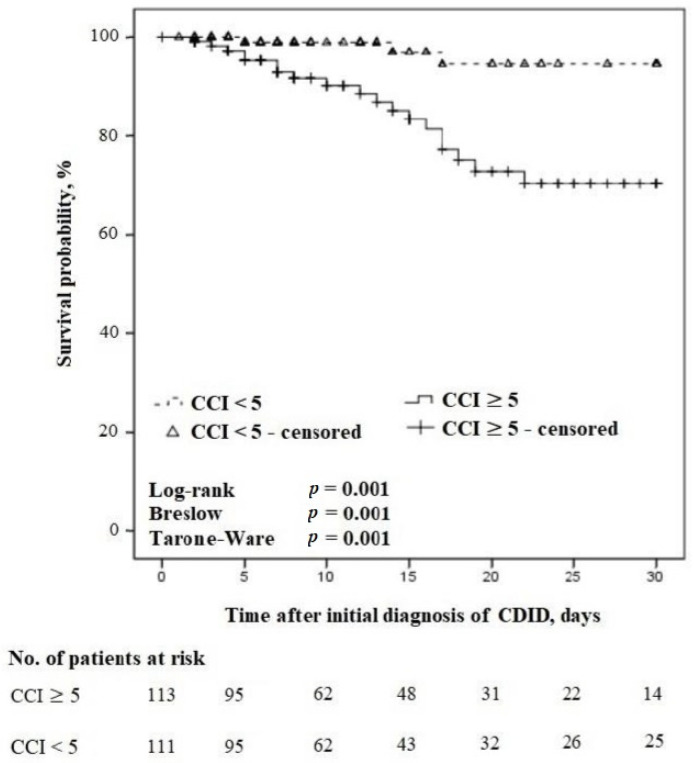
Kaplan–Meier survival curves for time to death in severe vs. non-severe comorbidities in patients with CDID receiving treatment concordant with the guidelines (censored at 30 days).

During the entire study period, a total of n = 113/224 (50.4%) patients receiving treatment concordant with the guidelines had a CCI score of ≥5. Figure 4 demonstrates the relationship between the comorbidities and 30-day survival of these patients.

In the 30-day follow-up, the 30-day all-cause mortality rate was n = 19/33 (57.6%) in patients receiving treatment concordant with the guidelines, and the CCI score was ≥5 compared with n = 3/28 (10.7%) in patients receiving treatment concordant with the guidelines and a CCI score of <5, *p* = 0.0002.

**Figure 5:**
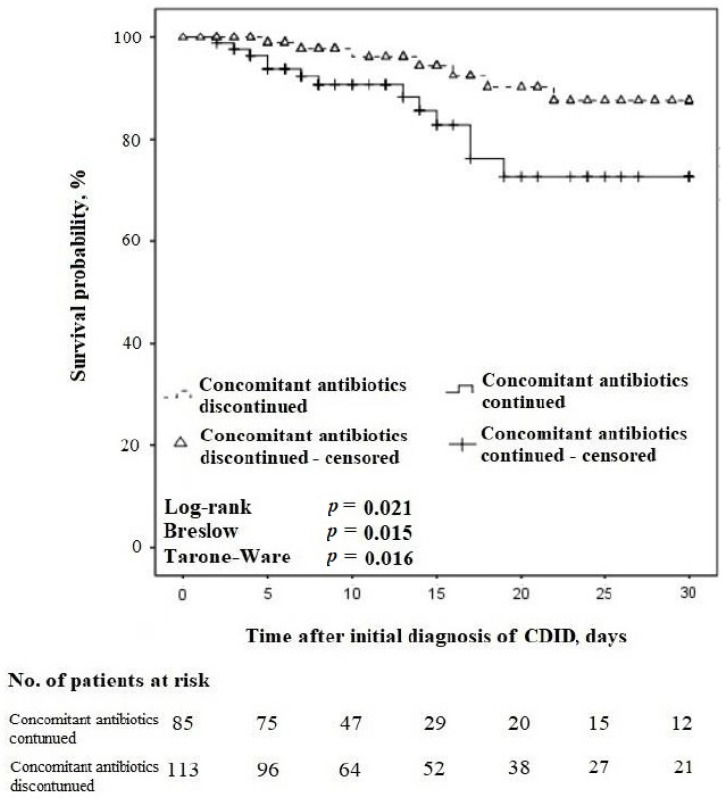
Kaplan–Meier survival curves for time to death regarding the continuation vs. discontinuation of concomitant antibiotics in patients with CDID also receiving treatment concordant with the guidelines (censored at 30 days).

Among CDID cases with treatment concordant with the guidelines, on the day of CDID diagnosis, n = 200/224 (89.3%) patients were using primary antibiotics for the treatment of other infections, and n = 86/200 (43.0%) continued the use of these antibiotics as concomitant ones along with the antibiotic for the treatment of CDID. The relationship between concomitant antibiotic use and the 30-day survival of these patients is presented in Figure 5.

In the 30-day follow-up, amidst the cases with CDID and treatment concordant with the guidelines, n = 53/61 (86.9%) patients were using primary antibiotics for the treatment of other infections on the day of CDID diagnosis, and n = 25/53 (43.4%) of them continued these antibiotics as concomitant. The 30-day all-cause mortality rate was n = 13/25 (52.0%) in patients continuing concomitant antibiotics compared with n = 7/28 (25.0%) in patients discontinuing them, *p* = 0.053.

### 3.6. CDID Cases before (Group 1) and after (Group 2) Integrating the 2017 Guidelines

CDID precipitating antibiotics have been used by n = 348/370 (94.1%) patients during the entire study period.

There were n = 197/370 (53.2%) patients in Group 1 compared with n = 173/370 (46.8%) in Group 2, and n = 187/197 (94.9%) used CDID precipitating antibiotics in Group 1 and n = 161/173 (93.1%) in Group 2, *p* > 0.05.

Table 3 demonstrates the dynamics in patient exposure to CDID precipitating antibiotics.

Regarding the dynamics, primary CDID was treated concordantly with vancomycin in n = 14/133 (10.5%) patients and with metronidazole in n = 119/133 (89.5%) Group 1 patients, compared with n = 43/91 (47.3%) and n = 48/91 (52.7%) in Group 2, *p* < 0.05. In Group 2, n = 91/173 (52.6%) of patients received treatment concordant with the guidelines.

## 4. Discussion

The highlights and novelties of our study were as follows. 1. Severe CDID cases with two severity criteria (leukocytes ≥ 15 × 109/L and serum creatinine level > 133 µmol/L) are associated with a refractory response when treated with metronidazole, non-concordantly with the guidelines. 2. Severe CDID cases with two vs. one severity criteria are associated with more CDID-related adverse outcomes (CDID-related ICU admission, CDID-related mortality) when treated with metronidazole, non-concordantly with the guidelines. We confirmed that CDID treatment non-concordant with the guidelines was associated with multiple adverse outcomes. In CDID cases with concordant treatment, severe comorbidities and continuation of concomitant antibiotics were associated with decreased 30-day survival also increased 30-day all-cause mortality and both were concluded as predictors of 30-day all-cause mortality. Unexpectedly, antibiotic optimization revealed no apparent impact on the CDID rate.

Following the primary objectives, our results revealed cases with treatment non-concordant with the 2017 guideline recommendations to be associated with all adverse outcomes analyzed in this study, including increased CDID-related ICU admission, CDID-related mortality, 30-day all-cause mortality and reduced 30-day survival. The findings of this report are similar to the ones published by Patel et al., who reported improper concordance with the previous 2010 guideline recommendations to result in the “under-treatment” of CDID and therefore be associated with elevated CDID-related Intensive Care Unit admission rate [32]. In addition, the study by Brown et al. found CDID treatment non-concordant with the 2010 guidelines to be associated with increased 30-day all-cause mortality [33]. Nonetheless, the clinical implementation of the 2017 guideline recommendations appears to be insufficient, as in our study, only 52.6% of CDID patients received CDID treatment concordant with the guidelines since 2017. Surprisingly, other authors found similar worrisome findings, with Patel et al. reporting that only 54%, and Brown et al. 51.7%, of CDID patients received treatment concordant with the guidelines [32,33]. In addition, Ebben et al. found that only 7% of patients with severe CDID received concordant treatment, compared with n = 20/103 (19.4%) in our report [34]. Various multidisciplinary strategies should be applied to achieve treatment concordance with the guidelines [35].

Despite many CDID cases being non-severe, as reported in our study (66.5%) and by other authors (60%), Clinical Practice Guidelines recommend the identification of precisely severe forms of CDID in order to optimize the treatment [12,36]. Bauer et al. established leukocytes > 15 × 10^9^/L and serum creatinine levels > 133 µmol/L at CDID diagnosis to be associated with treatment failure, and later, the detection of even a single value of those two criteria was accepted to be significant to diagnose the severe form of CDID, as also recommended by Clinical Practice Guidelines [12,37]. However, neither Bauer et al. nor the Clinical Practice Guidelines emphasize the importance of the contemporary presence of two (both) proposed severity criteria for treatment failure. As claimed within our primary objective and part of our hypothesis, we expected CDID cases defined by two severity criteria to be resistant to initial treatment when the provided treatment was non-concordant with the guidelines (due to the physician’s choice). Indeed, our results have shown treatment with non-concordant enteral metronidazole to be inferior in comparison with concordant enteral vancomycin, as metronidazole was associated with more refractory CDID in cases having two (both) diagnostic severity criteria. This appeared to be a novelty of our study. Based on various authors, the administration of vancomycin in CDID is associated with improved clinical recovery and lower 30-day mortality in the case of severe CDID compared to metronidazole [20,38]. Studies have shown that fecal metronidazole concentrations gradually decrease as *C. difficile* colitis resolves [39]. This prolonged gradual decrease in metronidazole concentration may be responsible for a period of insufficient treatment for the complete destruction of toxicogenic *C. difficile* bacteria after recovery from CDID, and activation of the residuum of unaffected *C. difficile* spores may later lead to the development of recurrent CDID [40,41]. These factors could have contributed to the consequences in patients with cogent severe form of CDID to be treated with just enteral metronidazole as was revealed in our study. Clinical Practice Guidelines recommended intravenous metronidazole in severe fulminant forms of CDID, though never as monotherapy, and always only as a part of combined treatment along with the crucially needed enteral vancomycin [12]. With enteral vancomycin, which is minimally or not absorbed from the gastrointestinal tract, the concentration in stool remains sufficiently high during the entire episode of CDID treatment [42]. It is also known that for CDID, intravenous vancomycin has no effect on the pathogen due to its insufficient secretion into the lumen of the gut. Only ~5% of this drug is being passed from the bloodstream into the gut with bile, while ~95% is eliminated unchanged via the kidneys [43]. For these reasons, Clinical Practice Guidelines have prioritized enteral vancomycin against metronidazole [12,44]. In relation to this, it should be noted that since 2021, the updated Clinical Practice Guidelines recommend fidaxomicin as an option of treatment for primary CDID, but vancomycin still strongly remains the treatment of choice or an acceptable alternative, as fidaxomicin could be up to ten times more expensive than vancomycin, and thus unavailable in some countries [44,45]. Moreover, some reports have also been published relating to the conflicting results in the cost-effectiveness of treatment with fidaxomicin compared with vancomycin [46,47,48,49]. In addition, according to published clinical and microbiological experience, the use of macrolides may be associated with the rapid development of bacterial resistance against all representatives of this class, though currently, the rate of *C. difficile* resistance to fidaxomicin is very low [50,51,52,53,54]. Thus, the benefit–cost ratio and potential future concerns about microbial resistance to fidaxomicin could render it inappropriate in clinical practice, and it often reduces the rate of fidaxomicin use for the treatment of CDID.

After the publication of the Clinical Practice Guidelines, Carlson et al. reported a study about the new definition of severe forms of CDID to be related to all-cause mortality [55]. However, they carried out their study with a total of both two and one criteria of severe-form cases to associate with all-cause mortality [55]. With the purpose of achieving a higher reliability of the results, based on our hypothesis, we deliberately made a cogent methodological choice to separately compare two criteria vs. one criterion, associating patient groups with CDID-related adverse outcomes. As part of our hypothesis claimed some outcomes in CDID to be more related to the decision of the clinician, we evaluated the endpoints in relation to the diagnostic degree of CDID severity by the presence of two (both) vs. one severity criteria in cases with treatment non-concordant with the guidelines. As expected, patients meeting two criteria receiving non-concordant treatment were at a higher risk of adverse outcomes, and, in fact, we determined they required more CDID-related Intensive Care Unit admissions and had higher CDID-related mortality compared with those meeting one criterion. These findings emphasize the importance of the clinical application of proposed severity criteria to improve the treatment of CDID, and suggest cases with two (both) CDID severity criteria to be “more severe” compared with cases meeting just one criterion [12]. This appeared to be a novelty of our study. Alongside that, we found the majority (78.3%) of our patients with severe CDID to be administered enteral metronidazole treatment. This may demonstrate the persisting inertia among clinicians to administer metronidazole as soon as the diagnosis of CDID has been established, without the differentiation of the severity of the disease with the purpose of achieving better outcomes, as recommended by the guidelines [12]. The administration of metronidazole in primary CDID has already been associated with more frequent cases of incomplete recovery [56]. Compared to our study, other authors used even more different criteria of CDID severity for association with mortality. Due to methodology particularities of using criteria noncoherent with the ones suggested by the 2017 Clinical Practice Guidelines, the results of these studies are difficult to compare with those of our study [12]. For example, Haubitz et al. reported the severe form of CDID due to the presence of ≥2 severity criteria, including leukocytosis, temperature ≥ 38.5 °C, hypoalbuminemia < 25 g/L and a shift in creatinine levels to be related to late, 60-day mortality [57]. In addition, the authors only reported the imprecise methodology of “usually” used doses of metronidazole, vancomycin and fidaxomicin for the treatment of CDID, thus not clearly differentiating the study sample in relation to the concordance of the administered treatment compared to our study [57]. Other authors reported associations between just one criterion from the two currently recommended, mostly leukocytosis > 15 × 10^9^/L, with outcomes such as treatment failure, recurrent CDID and 30-day all-cause mortality [19,58]. Thus, our hypothesis and the first objective have arisen from the need to identify patients with the most severe conditions meeting two criteria of the severe CDID form, including leukocytosis and serum creatinine level above the threshold. Concordance with Clinical Practice Guideline recommendations could be especially crucial in this group, as the lack of it was associated with treatment failure, as well as impaired CDID-related outcomes.

Comorbidities are common among patients with CDID and are generally reported as not only a risk factor for this disease, but also for its complications and even reduction in survival [59,60,61,62]. Thus, we evaluated the influence of comorbidities in patients with concordant treatment, and our study revealed severe comorbidities to be significantly related to reduced 30-day survival and increased 30-day all-cause mortality, as well as to be a predictor for 30-day all-cause mortality. In our report, a score of ≥5 was used to define severe comorbidities; as such, a threshold was previously recommended and already used in other publications [22,24,25]. Our results were similar to ones reported by Lee et al., who also associated a CCI score of ≥5 with decreased 30-day survival [23]. In addition, the previously mentioned authors also grouped patients in accordance with the feasibility of primary CDID treatment, and, interestingly, more than half of patients received non-concordant therapy for CDID [23]. In comparison, around two fifths of patients received non-concordant treatment in our study. The survival analysis itself allows for the visualization and continuous detection of ways in which a particular factor (comorbidities) influences the outcome (survival) [63]. In addition, it allows for the graphic visualization of patient censoring at certain time points [63]. Nonetheless, other authors are currently mostly reporting an association of comorbidities with just all-cause mortality in CDID. For example, Filippidis et al. demonstrated a higher CCI score to be associated only with a specific bundle of several adverse outcomes including all-cause mortality at day 10 and week 8 [64]. In comparison, we found the CCI score ≥ 5 to be directly related to 30-day all-cause mortality. The presented methodological differences between our study and the one by Filippidis et al. could have influenced the dissimilarity in the results, as in contrast to our report, the previously mentioned authors did not use a threshold for the CCI score and incorporated all-cause mortality only as a part of a bundle of outcomes to be evaluated at different time points [64]. In another study, Boeriu et al. found a slightly different threshold (>5) of the CCI score to be associated with the “risk of death”, but the authors did not specify if “death” was early or late compared to our results and other reports, where 30-day all-cause mortality was an objective to estimate [23,65]. In fact, a systematic review by Chakra et al. showed comorbidities to be a frequent risk factor for CDID, with complications needed for Intensive Care Unit admission because of fulminant colitis or shock, and to be related to worse outcomes [62]. In this paragraph, the reported associations of comorbidities with different adverse outcomes in CDID are due to methodological variations between the studies discussed above; thus, the direct comparison of their results is incompatible.

There is an absence of recommendations currently helping render clinical decisions for the feasible discontinuation of concomitant antibiotics in CDID. We concluded the continuation of concomitant antibiotics in around two fifths of the patients to be related to worse outcomes, even with treatment concordant with the guidelines. Indeed, these cases still appeared to have reduced 30-day survival and an increased 30-day all-cause mortality rate. It is possible that both the continuation of antibiotics and a poorer outcome may have occurred due to severe adjacent (non-*C. difficile*) infections still requiring antibiotic therapy to be continued. On the other hand, we cannot confirm the continuation of antibiotics to always be the underlying reason for this. Thus, the decision of concomitant antibiotic continuation is solely related to clinicians’ clinical knowledge and experience. Our results are similar to those published by Jin et al., who have also reported the continuation of concomitant antibiotics to increase 30-day mortality [16]. In addition, the previously mentioned authors reported a lower rate of continuation, at 33.4%, in comparison with 43.0% in our study [16]. The difference between the results of both studies could be attributed to distinct methodological differences, as Jin et al. incorporated a slightly different definition of a concomitant antibiotic [16]. Furthermore, the authors only included patients receiving metronidazole for the treatment of CDID, and have not differentiated whether CDID treatment feasibility was concordant with the guidelines as we did in our study; therefore, the results of our report could be more precise [12,16]. Another publication by Aguilar et al. reported the continuation of concomitant antibiotics rate in CDID to be even higher, at 74%, in comparison with our results, but the German authors did not associate it with mortality [66]. Reported variations in the rate of concomitant antibiotics continued in CDID may be related to an existing methodological gap in the definition of “concomitant antibiotic”. In addition, patients of different studies may have had very different varieties and severities of concurrent (concomitant) infections, needing the continuity of antibiotic therapy. Other associations regarding the continuation of concomitant antibiotics with the clinical trajectory of CDID have been published. Campbell et al. reported the use of concomitant antibiotics in CDID to be related to a longer duration of hospital stay, as well as higher treatment costs [67]. Finally, patients continuing the use of concomitant antibiotics after CDID diagnosis are at increased risk for recurrent CDID [28]. Thus, an immediate discontinuation of any unnecessary antibiotic therapy at the diagnosis of CDID is reasonably recommended whenever clinically justified [12]. In these cases, fidaxomicin should be a choice for CDID treatment, especially when concomitant antibiotics are continued, as the use of fidaxomicin is associated with higher efficacy of treatment and reduced risk of recurrent CDID [68].

According to the literature, antibiotic therapy is the main recognized risk factor for CDID [3,4,12,15]. In our study, as much as 94.1% of patients in total received antibiotics before *C. difficile* infection, and other researchers have reported similar results. Chang et al. reported antibiotic therapy rate before *C. difficile* to be 93% in comparison with 94.7%, as reported by Hung et al. [69,70]. Therefore, the use of CDID precipitating antibiotics remains the dominating risk factor. In addition to the latter statement, the overall awareness of this predominantly nosocomial infection and its etiology appears to be increasing among clinicians. 

Thus, one of the objectives of this study was to investigate the stewardship of antibiotics prior to CDID, where we expected some reduction in broad-spectrum antibiotic use. Indeed, as multiple sources have reported a significantly higher risk of CDID with the use of cephalosporins, we noted that second-generation and non-antipseudomonal third-generation cephalosporin use has significantly decreased over time [71,72,73,74,75]. We expect this to be related to better diagnosis of infections and improved knowledge in the use of antibiotics among clinicians in general, policies of limited access for antibiotics in population, etc. In our study, we also discovered a reliable increase in the use of penicillin-class antibiotics and macrolides in dynamics, which is more ecological in terms of reduced negative effect on the human microbiota due to the narrower antimicrobial spectrum of penicillin, as well as due to the mainly bacteriostatic action of macrolides [76,77,78,79]. Treatment with antibiotics, besides infection control, always leads to the suppression of the natural gut microbiota, reduces its diversity and induces dysbiosis development, which is an essential risk factor of CDID [2,80]. A variable grade of CDID risk caused by different classes of antibiotics could be explained by the differences in the antimicrobial spectrum and the susceptibility of intestinal bacteria to the certain antibiotic [75,81]. Among antibiotics associated with the highest risk of CDID, cephalosporins usually have a broad antimicrobial spectrum; therefore, they are more prone to induce significant gut dysbiosis, creating an excellent environment for the growth of colonizers such as *C. difficile* bacteria [74,75,81]. Thus, antibiotic-induced dysbiosis may persist with an increased risk of CDID for even three months after the discontinuation of antibiotic therapy [26]. However, despite the reduced use of cephalosporins before CDID increasing concordant vancomycin use for primary CDID treatment as part of ongoing antibiotic optimization, the incidence of CDID did not appear to decrease in dynamics. This might be related to better ability among clinicians to recognize CDID. Moreover, regardless of the differences in the antimicrobial spectrum of many commonly administered antibiotics, all of them appear to always be significantly associated with CDID in comparison with cases without antibiotic use [82].

Guidelines significantly facilitate clinical decisions, and failure to comply with treatment recommendations is associated with treatment failure, as well as CDID-related Intensive Care Unit admission and CDID-related mortality [12,32]. Nonetheless, it remains necessary for clinicians to approach all patients individually with the comprehension that ultimately, medicine itself is “Science and Art”, challenging the decisions of precipitating and concomitant antibiotic use, especially in severe CDID [83].

### Study Novelties and Limitations

Although the current study is retrospective and single-center, it provided information about the importance of following guideline recommendations for better outcomes, as well as associations of clinical and laboratory characteristics with different outcomes in patients with CDID. It also included a novel finding that two-criteria-defined severe CDID cases had more a frequent refractory response when treatment was non-concordant with the 2017 Clinical Practice Guideline recommendations [12]. Our study also revealed that severe cases defined by two severity criteria with non-concordant treatment were associated with more frequent CDID-related adverse outcomes (thus, “more severe”) when compared with severe CDID cases defined by only one criterion. Regarding the applicability and generalization of our data, they were collected from medical departments of various profiles of the largest referral university hospital in Lithuania; thus, we consider our results to reflect the CDID profile of the country. In addition, many inpatients with more complicated conditions from regional hospitals are always referred to our hospital from various parts of the country. Finally, our report could be used as a platform for larger prospective future studies.

Some limitations of our study need to be addressed. Fecal *C. difficile* glutamate dehydrogenase and nucleic acid amplification testing, which are used for diagnosis together with A and B toxins to increase diagnostic sensitivity and specificity, were not performed at our hospital during the study period [84,85,86,87]. Due to this limitation, the actual incidence of CDID and the number of patients eligible for the study could have been higher. The retrospective nature of our study also limited the eligibility for a higher number of patients to be included in the 30-day follow-up. A greater size of the 30-day follow-up group would have likely resulted in a significant (in spite of only clinical relevance) increase in the 30-day all-cause mortality due to the continuation of concomitant antibiotics. Nevertheless, it should be noted that the total sample size of our study still met the study size requirement. Moreover, the retrospective nature of the data collection may have the limitation of a precise trace back of antibiotics used before primary CDID. However, the already-recorded information about precipitating antibiotics is sufficient for the trajectories of the use of antibiotics in the past. Furthermore, the ribotypes of *C. difficile* in the study could not have been identified and reported; however, according to Lithuanian National data, approximately 60–70% of all tested *C. difficile* strains from the country’s hospitals belong to the hypervirulent 027 ribotype [88]. Thus, findings relating non-hypervirulent *C. difficile* strains may differ from the results of our study. Finally, within the period of this study, the use of fidaxomicin was also almost always unavailable (used sporadically), as the drug was yet to be registered. 

## 5. Conclusions

Our results suggest that CDID treatment non-concordant with the guidelines was associated with various adverse outcomes. Therefore, concordance with evidence-based (guidelines) recommendations should be mandatorily improved for better outcomes in CDID patients. Additionally, in severe CDID cases defined by leukocytes ≥ 15 × 10^9^/L and serum creatinine level > 133 µmol/L (>1.5 mg/dL), guideline-concordant enteral vancomycin should be used to avoid a refractory response, as metronidazole use (non-guideline concordant) was associated with worse CDID-related outcomes. Severe comorbidities were always associated with worse outcomes, even in cases implementing guideline-concordant treatment. The continuation of concomitant antibiotic treatment administered at CDID onset needs to be reasonably justified, deescalated or stopped, as it was related to worse outcomes. Interestingly and unexpectedly, antibiotic optimization had no apparent impact on the CDID development rate.

## Figures and Tables

**Table 1:** Characteristics of patients with CDID.

General Characteristics	
Age, years (Q1; Q3)	67.0 (54.0; 77.0)
Immunocompromised patients, n (%)	205 (55.4)
Serum creatinine, µmol/L (Q1; Q3)	140.7 (76.0; 124.0)
Leucocytes, n × 10^9^/L (Q1; Q3)	10.1 (3.8; 13.5)
Hospital departments diagnosing CDID	
Medical, n (%)	299 (80.8)
Surgical, n (%)	56 (15.1)
Intensive Care Unit, n (%)	15 (4.1)

**Table 2:** Predictors of 30-day all-cause mortality in CDID cases with concordant treatment.

Variable	OR for 30-Day All-Cause Mortality	95% CI	*p*-Value
CCI score	1.76	1.2–2.5	0.003
Continuation of concomitant antibiotic	3.2	1.02–10.4	0.047
Leucocyte count, n × 10^9^/L	1.09	0.9–1.2	0.07
Creatinine level, µmol/L	1.003	0.9–1.03	0.7
Presence of immunosuppression	0.5	0.1–2.4	0.4

OR—odds ratio; CCI—Charlson Comorbidity Index; CI—confidence interval.

**Table 3:** Classes of CDID precipitating antibiotics.

Class of Antibiotic	Group 1	Group 2	*p*-Value
Second-generation cephalosporin, n (%)	149 (75.6)	102 (59.0)	0.001
Non-antipseudomonal third-generation cephalosporin, n (%)	18 (9.1)	4 (2.3)	0.007
Penicillin with beta-lactamase inhibitor, n (%)	42 (21.3)	59 (34.1)	0.007
Macrolide, n (%)	4 (2.0)	13 (7.5)	0.013

## Data Availability

The data presented in this study are available on request from the corresponding authors.

## Supplementary Materials

The following supporting information can be downloaded at https://www.mdpi.com/article/10.3390/antibiotics13010051/s1, Supplementary Material S1. PICO chart of the study objectives. Supplementary Material S2. STROBE checklist.
