# Supplementary material for: Improving Clostridioides difficile Infectious Disease Treatment Response via Adherence to Clinical Practice Guidelines
Source: Antibiotics (Basel). 2024 Jan 4;13(1):51. doi: 10.3390/antibiotics13010051 (PMC10812669; doi:10.3390/antibiotics13010051)
Supplement: Supplementary file 1 [file antibiotics-13-00051-s001.zip › Supplementary Material S1. PICO chart of the study objectives. F.pdf]

# Supplementary Material S1. PICO chart of the study objectives

**Primary objective 1.**

**P:** All cases with diarrhea, confirmed *C. difficile* A and/or B toxins in stools, severe also non-severe CDID, and concordant also non-concordant treatment
**I:** Concordant with Guidelines treatment
**C:** Non-concordant with Guidelines treatment
**O:** CDID–related Intensive Care Unit admission, CDID–related mortality, 30-day survival, 30-day all-cause-mortality.

**Primary objective 2.**

**P:** All cases with diarrhea, confirmed *C. difficile* A and/or B toxins in stools, blood leucocyte count $< 15 \times 10^9$/L, creatinine serum level $\leq 133$ µmol/L, and concordant also non-concordant treatment
**I:** Concordant with Guidelines treatment
**C:** Non-concordant with Guidelines treatment
**O:** CDID–related Intensive Care Unit admission, CDID–related mortality

**Primary objective 3.**

**P:** Cases with diarrhea, confirmed *C. difficile* A and/or B toxins in stools, blood leucocyte count $\geq 15 \times 10^9$/L, creatinine serum level $> 133$ µmol/L, and concordant also non-concordant treatment
**I:** Concordant enteral vancomycin treatment (Group V)
**C:** Non-concordant enteral metronidazole treatment (Group M)
**O:** Treatment failure (refractory CDID)

**Primary objective 4.**

**P:** Patients with diarrhea, confirmed *C. difficile* A and/or B toxins in stools, and non-concordant enteral metronidazole treatment (Group M)
**I:** Blood leucocyte count $\geq 15 \times 10^9$/L, **and** creatinine serum level $> 133$ µmol/L
**C:** Blood leucocyte count $\geq 15 \times 10^9$/L, **or** creatinine serum level $> 133$ µmol/L
**O:** CDID–related Intensive Care Unit admission, CDID–related mortality

**Secondary objective 1.**

**P:** Patients with diarrhea, confirmed *C. difficile* A and/or B toxins in stools, severe also non-severe CDID, and concordant treatment
**I:** CCI score $\geq 5$
**C:** CCI score $< 5$
**O:** 30-day survival, 30-day all-cause mortality

**Secondary objective 2.**

**P:** Patients with diarrhea, confirmed *C. difficile* A and/or B toxins in stools, severe also non-severe CDID, and concordant treatment

**I:** Continuation of concomitant antibiotics

**C:** Discontinuation of concomitant antibiotics

**O:** 30-day survival, 30-day all-cause mortality

**Secondary objective 3.**

**P:** Patients with diarrhea, confirmed *C. difficile* A and/or B toxins in stools, severe also non-severe CDID, and concordant also non-concordant treatment

**I:** The use of CDID precipitating broad-spectrum antibiotics also vancomycin for primary CDID treatment in 2011-2016 (Group 1)

**C:** The use of CDID precipitating broad-spectrum antibiotics also vancomycin for primary CDID treatment in 2017-2020 (Group 2)

**O:** Rate of CDID
